# Wonderful Coal Products

**Published:** 1890-09

**Authors:** 


### WONDERFUL COAL PRODUCTS.

Though pit-coal has been known for some hundreds of years, the discovery of its numberless products is confined to the present century. Illuminating gas was unknown 100 years ago. Petroleum has been in use only about forty years, and it is scarcely more than fifty since some one discovered that stone coal was inflammable. Nearly all the other products derived from soft coal have been discovered and applied in the interest of science or of fraud within the last twenty-five years. The first thought in regard to coal is that it is made to give heat or warmth; the next that one of its principal uses is to illuminate. But there are obtained from it the means of producing over 400 shades of colors, among the chief of which are saffron, violet-blue and indigo. There is also obtained a great variety of perfumes—cinnamon, bitter almonds, queen of the meadows, clove, wintergreen, anise, camphor, thymol (a new French odor), vanaline and heliotropine. Some of these are used for flavoring. Among the explosive agents whose discovery has been caused by the war spirit of the last few years in Europe are two called dinitrobenzine or bellite, and picrates. To medicine coal has given hypnone, salicylic acid, naphtol, phenol and antipyrine. Benzine and naphthaline are powerful insecticides. There have been found in it ammoniacal salts, useful as fertilizers, tannin, saccharine (a substitute for sugar), the flavor of currants, raspberry and pepper, pyrogallic acid and hydroquinone, used in photography, and various substances familar or unfamiliar, such as tar, resin, asphaltum, lubricating oils and the bitter taste of beer. By means of some of these we can have wine without the juice of the grape, beer without malt, preserves without either fruit or sugar, perfumes without flowers, and coloring matters without the vegetable or animal substances from which they have been hitherto chiefly derived.—*Detroit Free Press.*

